# [Corrigendum] Macrophage-derived foam cells impair endothelial barrier function by inducing endothelial-mesenchymal transition via CCL-4

**DOI:** 10.3892/ijmm.2026.5824

**Published:** 2026-04-03

**Authors:** Ying Yang, Nian-Sang Luo, Ru Ying, Yong Xie, Jia-Yuan Chen, Xiao-Qiao Wang, Zhen-Jie Gu, Jing-Ting Mai, Wen-Hao Liu, Mao-Xiong Wu, Zhi-Teng Chen, Yong-Biao Fang, Hai-Feng Zhang, Zhi-Yi Zuo, Jing-Feng Wang, Yang-Xin Chen

Int J Mol Med 40: 558-568, 2017; DOI: 10.3892/ijmm.2017.3034

Following the publication of the above article, an interested reader drew to the authors' attention that, in Fig. 3 on p. 563, the cell morphological images shown for Fig. 3E and F, representing the results for M2a- and M2c-derived foam cells respectively, contained an overlapping section, such that data which were intended to show the results from differently performed experiments had apparently been derived from the same original source.

The authors were able to consult their original data, and realized that the data panel shown to represent Fig. 3E had inadvertently been included in this figure erroneously. The revised version of Fig. 3, now showing the correct data for Fig. 3E, is shown on the next page. The authors can confirm that the error associated with this figure did not have any significant impact on either the results or the conclusions reported in this study, and all the authors agree with the publication of this Corrigendum. The authors are grateful to the Editor of *International Journal of Molecular Medicine* for allowing them the opportunity to publish this Corrigendum; furthermore, they apologize to the readership of the Journal for any inconvenience caused.

## Figures and Tables

**Figure 3:**
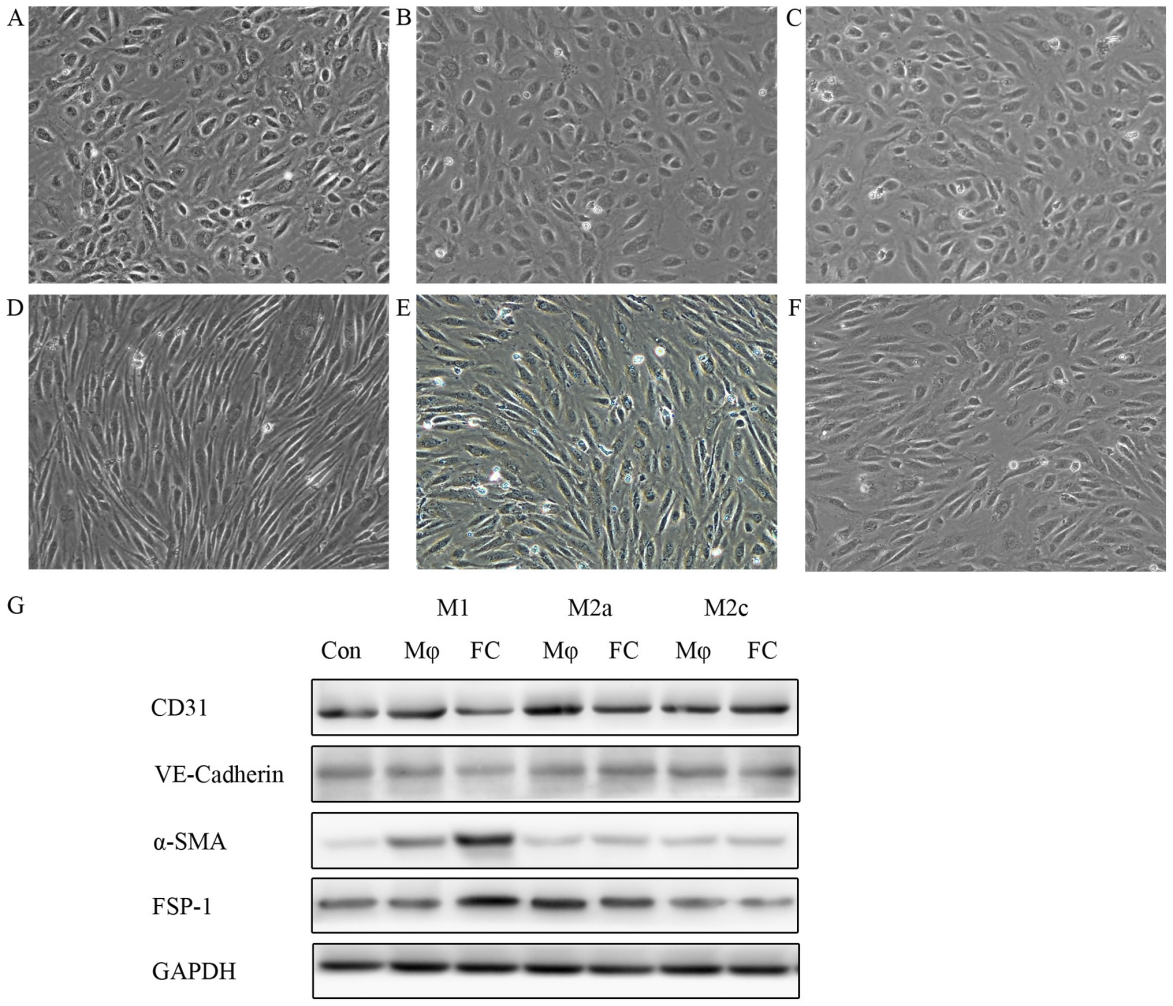
Conditioned medium from M1-FCs stimulates HAECs to undergo EndMT *in vitro*. HAECs were treated with conditioned medium from different phenotypic macrophages: conditioned medium from (A) M1, (B) M2a and (C) M2c macrophages and (D) M1, (E) M2a and (F) M2c-derived FCs at 1:1 ratio with EBM-2 for 6 days. HAECs treated with conditioned medium from M1-FCs lost the typical cobblestone-like morphology and gained a spindle-like appearance. (G) Western blot analysis of endothelial and mesenchymal cell surface markers demonstrated that HAECs incubated with conditioned medium from M1-FCs underwent EndMT. M1-FCs, M1 macrophage-derived foam cells; Con, control; Mφ, macrophage; FC, foam cell; VE-cadherin, vascular endothelial cadherin; α-SMA, α-smooth muscle actin; FSP-1, fibroblast-specific protein-1; EndMT, endothelial mesenchymal transition.

